# Molecular evolution and functional divergence of eukaryotic translation initiation factor 2-alpha kinases

**DOI:** 10.1371/journal.pone.0194335

**Published:** 2018-03-14

**Authors:** K. Hari Krishna, Muthuvel Suresh Kumar

**Affiliations:** 1 Centre for Bioinformatics, Pondicherry University, Kalapet, Pondicherry, India; 2 Department of Biotechnology, Vignan's Foundation for Science, Technology & Research (VFSTR) University, Vadlamudi, Andhra Pradesh, India; Michigan State University, UNITED STATES

## Abstract

Eukaryotic translation initiation factor 2-alpha kinase (EIF2AK) proteins inhibit protein synthesis at translation initiation level, in response to various stress conditions, including oxidative stress, heme deficiency, osmotic shock, and heat shock. Origin and functional diversification of EIF2AK sequences remain ambiguous. Here we determine the origin and molecular evolution of EIF2AK proteins in lower eukaryotes and studied the molecular basis of divergence among sub-family sequences. Present work emphasized primitive origin of EIF2AK4 sub-family gene in lower eukaryotes of protozoan lineage. Phylogenetic analysis supported common origin and sub-family based classification of EIF2AKs. Functional divergence studies across sub-families revealed several putative amino acid sites, which assist in altered protein interactions of kinase domains. The data can facilitate designing site-directed experimental studies aiming at elucidating diverse functional aspects of kinase domains regarding down-regulation of protein synthesis.

## Introduction

The eIF2α kinases are a family of four distinct serine-threonine kinases, EIF2AK1, EIF2AK2, EIF2AK3 and EIF2AK4, referred to as HRI (Heme-Regulated Inhibitor), PKR (Protein Kinase double-stranded RNA-dependent), PERK (protein kinase R (PKR)-like endoplasmic reticulum kinase), and GCN2 (General Control Nonderepressible2) respectively[1]. Mammalian EIF2AKs have a conserved Kinase Domain (KD) but distinct regulatory domains that mediate activation by diverse stress signals[2]. These are stress-activated protein kinases that downregulate protein synthesis and simultaneously up-regulated transcriptional activators at the translational level. This dual response reduces cellular protein synthesis while reprogramming transcription to favor gene products with functions in stress management[3]. These kinases carry phosphorylation of translation initiation factor 2 (eIF2α) at the Ser51 residue of α-subunit. eIF2 binds GTP to onset initiation pathway delivering methionyl initiator tRNA to 40s ribosomal subunit, which produces the 43S pre-initiation complex. Initiator tRNA recognizes AUG codon, activating eIF2B, an exchange factor for guanine nucleotide, which hydrolyzes GTP releasing eIF2GDP from 40S subunit for recycling back to eIF2GTP. Phosphorylated eIF2 is an inhibitor of eIF2B, which reduces the concentration of eIF2 GTP inhibiting translation initiation. Reduced eIF2GTP levels stimulate translation of GCN4 mRNA in yeasts and ATF4 mRNA in mammals, which encode transcriptional activators of stress genes[4–6].

EIF2AK1 or HRI maintains heme levels in the cell by playing a key role in the synthesis of globin genes and promoting erythroid precursors survival[7]. Proposed model suggests that HRI binds to Heme in its N-terminal end. This triggers an intermolecular autophosphorylation generating HRI-HRI component, which senses Heme concentrations. Further autophosphorylation events activate HRI in heme deficiency conditions and it proceeds to phosphorylate eIF2α. Protein makes up of N-terminus and C-terminus regions flanking a central KD. N-terminus and KD regions harbor binding sites for heme. N-terminus contains a stable heme-binding site, needed for heme regulation of HRI while KD harbors reversible heme-binding site[8]. Flanking histidine residues align heme molecule at both sites.

EIF2AK2 or PKR phosphorylates eIF2α in response to viral infection, thereby blocking translation of viral mRNAs and promoting apoptosis[9]. PKR plays a key role in activation response to diverse signals, such as oxidative and endoplasmic reticulum (ER) stress, cytokine signaling and growth factors[10–13]. PKR’s N-terminal region contains a regulatory double-stranded RNA binding domain (dsRBD) consisting of two dsRNA binding motifs that are separated by an unstructured linker region[14]. C-terminal contains a KD, which bears catalytic functions of protein and has crucial dimerization interface for activation of PKR. Binding of viral dsRNA to PKR induces dimerization of C-terminal KD[15,16]. Autophosphorylation events stabilize resulting dimer, which phosphorylates eIF2α.

Misfolded proteins accumulate in the endoplasmic reticulum (ER) which creates a phenomenon of ER stress activating EIF2AK3 or PERK[17]. PERK is an ER transmembrane protein consisting of the N-terminal region important for dimerization, regulation, and association with ER chaperone, immunoglobulin binding protein (BiP) and HspA5, while the C-terminal region is cytosolic and contains KDs with autophosphorylation sites[18]. Misfolded proteins induce BiP bound PERK in the active state to dissociate and allow dimerization and autophosphorylation activating PERK[19,20]. Additional proposed activation mechanism involves PERK functions as a sensor of ER calcium levels[21,22]. Reduction in ER calcium levels activates PERK.

EIF2AK4 or GCN2 is activated by deprived levels of amino acids and by deprived levels of glucose in cells. GCN2 has a more complex structure than PERK and PKR[23,24]. It harbors the typical KD; it also harbors a pseudo-KD and a histidyl-tRNA synthetase (HisRS) related domain, which has a higher affinity for uncharged tRNAs. Binding of uncharged transfer RNAs (tRNAs) to HisRS domain of protein dimerizes and autophosphorylates protein as other eIF2α kinases which activate GCN2[25]. Activated GCN2 phosphorylates eIF2α, which causes a general inhibition of translation initiation and simultaneously favors selective translation of some mRNAs containing short open reading frames (ORFs)[26]. These encode bZIP (basic leucine zipper) transcription factors that induce expression of genes, which aid cellular adaptation to amino acid deprivation[27,28].

EIF2AKs share a common physiological role of phosphorylating eIF2α substrate that acts as an initiator of the translation process. EIF2AKs recognizes diverse signals at cellular levels due to their diverse signaling domains. KD is a key factor aiding in dimerization and in eIF2α phosphorylation, shows conservation across four sub-families and across species. An understanding divergence of these sequence sub-families provides insights into substrate specificity of kinases. We used bioinformatics and computational approaches to identify novel annotated EIF2AK sequences in lower organisms, address the evolutionary and phylogenetic relationship of EIF2AKs across four sub-families, analyze sites responsible for the evolution of functional divergence of EIF2AK sub-families.

## Methodology

### Identification of EIF2AK sequences

Annotated EIF2AK sequences were retrieved from UniProt (http://www.uniprot.org/)[29]. Hidden Markov Model (HMM) is built with HMMBUILD program of HMMER3 (http://hmmer.janelia.org/)[30] using above retrieved sequences. HMM profile is invoked as a query for HMMSEARCH program to search 250 vertebrate proteomes retrieved from Ensembl[31], Joint Genome Institute (JGI) (http://www.jgi.doe.gov/)[32] and NCBI genome (http://www.ncbi.nlm.nih.gov/genome/) databases[33]. Simultaneously, a search of NCBI Protein (http://www.ncbi.nlm.nih.gov/protein) sequence database using keyword search “EIF2AK” and sequences are manually curated. Identical sequences in the retrieved sequences were removed using CD-HIT software[34] with default tuple size and a cutting threshold of sequence identity of 95%.

### Multiple sequence alignment and phylogenetic tree construction

A multiple sequence alignment (MSA) is obtained based on final dataset of EIF2AK sequences trimmed to conserved KDs. Local alignment on sequences is performed using MAFFT software[35] (http://mafft.cbrc.jp/alignment/software/), with PAM as scoring matrix to align functional motifs of diversified sequences. Based on quality of alignment unaligned or packed columns in MSA were removed manually using Jalview[36] software retaining good quality columns. Phylogenetic trees were built based on Bayesian Inference using MrBayes software version 3.2[37] using mixed amino acid model. Markov Chain Monte Carlo (MCMC) analysis is utilized to approximate posterior probabilities of phylogenetic trees. An analysis is executed for 20 million generations with sampling of every hundredth tree. A stop rule with a stop value of 0.05 is applied to terminate MCMC run and to assess convergence of MCMC. Sump value of 0.25 discards first 25% of sampled trees at burnin period after MCMC’s had converged and substitution model parameters, including mean, mode, and 95% credibility interval of each parameter were estimated. Sumt command is used to build a consensus tree from remaining 75% of sampled trees using 50% majority rule. Phylogenetic trees were viewed using Dendroscope 3 program[38].

### Analysis of gene structure and gene order

Gene Structure Display Server (GSDS)[39] (http://gsds.cbi.pku.edu.cn/) is used to analyze gene structure. Representative gene sequences of each phyla of animal kingdom are submitted to GSDS server for generating visualization of gene features such as composition and position of exons and introns. Cinteny server (http://cinteny.cchmc.org/)[40] is used to analyze synteny and evolutionary distances in terms of genome rearrangements for selected organisms. Cinteny server allows comparing multiple genomes and performing sensitivity analysis for synteny block detection and for subsequent computation of reversal distances. Users can interactively browse gene blocks conserved in multiple genomes using Cinteny server, to facilitate genome annotation and validation of assemblies for newly sequenced genomes, and to construct and assess phylogenetic trees.

### Comparison of selection pressure

Evolutionary pressure of substitution rates is calculated for selected EIF2AK sequences from different phyla of animal kingdom to analyze nature of substitutions in these sub-families. PAL2NAL (http://www.bork.embl.de/pal2nal/) server[41] is utilized to generate a codon based alignment of DNA sequences based on MSA of protein sequences. Resulting codon alignment is used in CODEML program of the PAML package[42] to calculate rate of non-synonymous and synonymous substitution (ω = dN/dS). An ω value >1 with increased dN value indicates rapid amino acid substitution implying positive selection on gene, and in contrast ω value < 1 indicates elimination of disadvantage replacements, suggesting purifying selection. If dN = dS (or ω = 1) gene evolves neutrally by silent or missense mutations with no significant alterations in gene function.

### Estimation of functional divergence

Functional divergence between four sub-families of EIF2AK sequences and related kinase sequences is inferred by type I and type II divergence analyses utilizing Diverge 3.0[43]. Type I functional divergence suggests heterogeneous evolutionary rates between duplicated genes, while type II functional divergence suggests radical changes to biochemical properties like charge (positive or negative), nature (hydrophilic/hydrophobic), between duplicates. Site-specific estimation of posterior probability of radical changes related to type II functional divergence is performed to assess probable regions and shifts of biochemical properties between paralogous groups[44].

## Results and discussion

### Identification and classification of EIF2AK sequences

852 sequences in total consisting of annotated and unannotated sequences were identified using HMMSEARCH of sequenced genomes and NCBI sequence keyword based searching. Further classification of sequences at sub-family level identified a set of 169 EIF2AK1, 148 EIF2AK2, 206 EIF2AK3 and 329 EIF2AK4 sequences. EIF2AK1 and EIF2AK3 sub-family sequences show their origin from Nemathelminthes and Platyhelminthes. EIF2AK2 shows a later evolutionary origin in phylogenetic lineage with its origin from higher chordates Osteichthyes (Bony fish). EIF2AK4 occurs down evolutionary lineage with occurrence from lower eukaryotic species like Viridiplantae, which comprises of lower algal forms inferring its primitive ancestry in EIF2AK family. All EIF2AK sub-families show a continuous distribution by their presence in higher organisms across evolutionary order. HMMSEARCH of bacterial model organisms provide insight to prokaryotic origin of EIF2AKs from Protein kinase B (PknB) a Serine/threonine-protein kinase (STPK). PknB shares striking similarity in protein fold, catalytic machinery and kinase regulation mechanism with eukaryotic STPKs[45–47]. S1 Table shows distribution of EIF2AKs across phyla along with reference number.

### Domain organization of EIF2AK family

**Fig 1:**
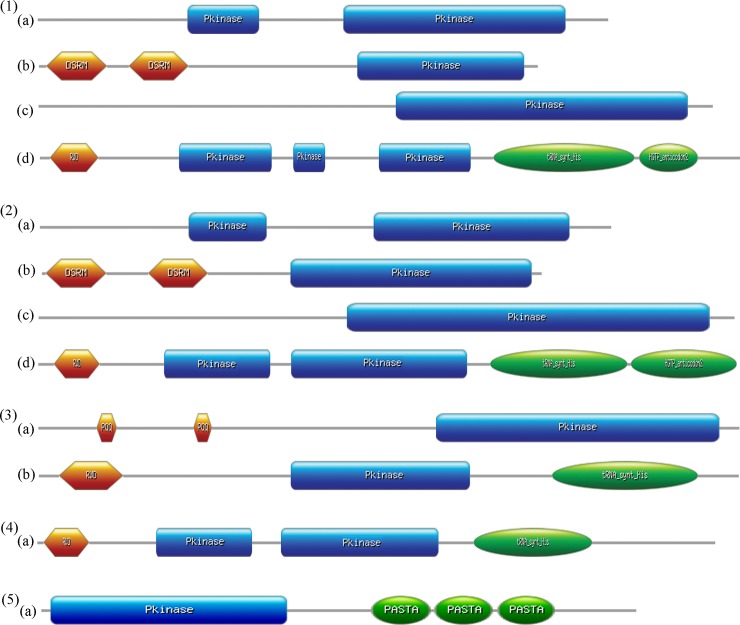
Domain organization of EIF2AKs. Domain organization of the representative sequences from across the evolutionary lineage is shown. (1)(a) to (1)(d) show the domains of *Homo sapiens’* EIF2AK1, EIF2AK2, EIF2AK3, EIF2AK4. (2)(a) to (2)(d) show the domains of *Xenopus tropicalis’* EIF2AK1, EIF2AK2, EIF2AK3, and EIF2AK4. (3)(a), (3)(b) show the domains of EIF2AK3 and EIF2AK4 of *Musca domestica*. (4)(a) shows the domain of EIF2AK4 from *Saccharomyces cerevisiae*. (5)(a) shows the domain of STPK from *Bacillus subtilis*.

EIF2AK family proteins show a varied domain organization majorly at signaling domain due to a wide range of activation signals they recognize (Fig 1). EIF2AK1 has a partial KD followed by a functional full-length KD. EIF2AK2 in humans has two Double-Stranded RNA-binding Motifs (DSRM), which contribute to recognition of dsRNA of viral origin and a functional KD. EIF2AK3 has a single KD with heme recognition motif, which is sensitive to heme levels. In lower non-chordates, EIF2AK3 has a dimerized Pyrrolo-quinoline quinone (PQQ) domain that aids in electron transfer reactions like transfer of phosphate moiety due to relatively high redox potentials of PQQ/PQQH_2_ couple[48]. EIF2AK4 has a complex structure with two nonfunctional pseudo KDs followed by a functional KD. It also harbors a histidyl-tRNA synthetase (HisRS)-related domain, which binds uncharged tRNAs. RWD domain at N terminal is located in RING finger and WD repeat containing proteins and DEXDc-like helicases. It also harbors a HGCTP anticodon domain, which aids in down regulation of translation process in stress conditions. Domain organization of EIF2AK4 shows a similar domain architecture in lower eukaryotes with loss of partial KDs. Domain organization of bacterial PknB kinase shows a typical KD, which plays a critical role in signal transduction pathway that regulates cell growth, cell shape and cell division[49–51]. Three PASTA (Penicillin-binding protein and Serine/Threonine kinase Associated) domains activate protein by dimerization of KD by allosteric mechanism and trigger auto phosphorylation events[52,53].

### Multiple sequence alignment and phylogenetic analysis

**Fig 2:**
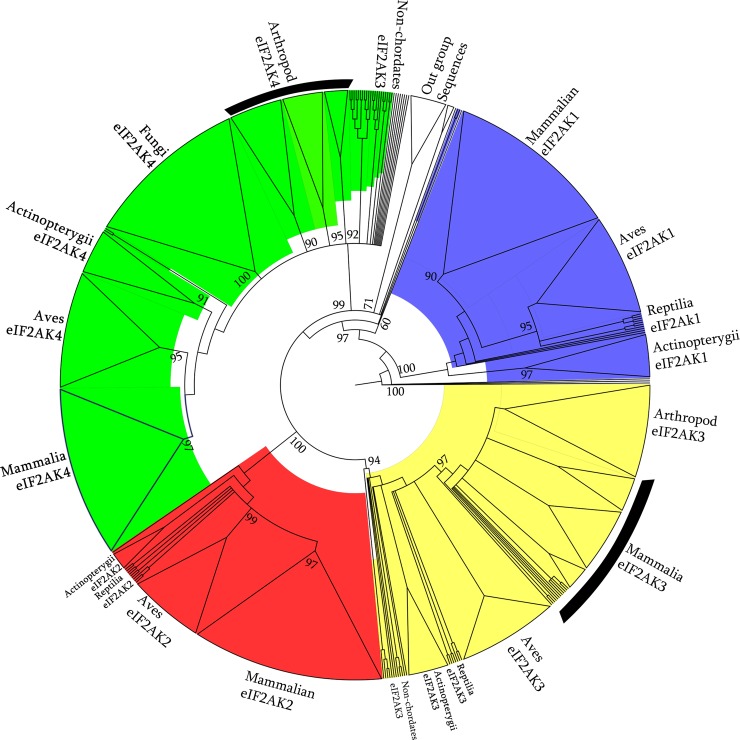
EIF2AK phylogenetic analysis. Phylogenetic tree constructed by Bayesian method, which shows the branching of EIF2AK sequences into closely related subgroups. Four sub-families EIF2AK1, EIF2AK2, EIF2AK3 and EIF2AK4 are highlighted with blue, red, yellow and green colors respectively indicating a definite cluster. Out-group sequences of NEK2, TOPK, TTK, WEE1 and bacterial STPK clustered as distinct groups. Clusters of each distinct phyla are supported by confidence values at branching nodes.

To investigate evolutionary relationships of identified EIF2AK sequences we adopted Bayesian methods of phylogenetic reconstruction with identified 852 EIF2AK family sequences and outgroup sequences of closely related families via NEK2, TOPK, TTK, WEE1 and bacterial STPK. The inferred Bayesian tree topology shows an unrooted tree with a four closely grouped clades, supported by a good confidence value of 100 percent representing clustering of four sub-families of EIF2AK sequences (Fig 2). Outgroup sequences cluster into definite groups showing similarity among EIF2AK sequences compared to outgroup sequences. These phylogenetic groupings are consistent with aforementioned identity based methods of hmm and BLAST based classification methods. This phylogenetic classification of sequences is also useful in classification of highly ambiguous sequences. Four clades branched out were further split into divergent sub-clades, which are organized by evolutionary hierarchy of taxa present at each node. EIF2AK1 clade shows nematodes, arthropod, molluscans and hemichordates closer to node showing primitive origin of gene in lower organisms. Higher chordates from fish, reptiles and mammals group into three distinct subgroups. EIF2AK2 sequences show only presence of chordates that are further divided into discrete two sub groups, one consisting of sequences of Reptiles and Aves, and other group consisting of mammals, highly evolved chordates. EIF2AK3 sequences show a widespread distribution of subclades from Platyhelminthes to higher chordates. EIF2AK4 sequences show a highly clustered sequences which consist of lower evolutionary fungal sequences, other making up of Reptiles, Aves, and third cluster consisting of Mammals.

**Fig 3:**
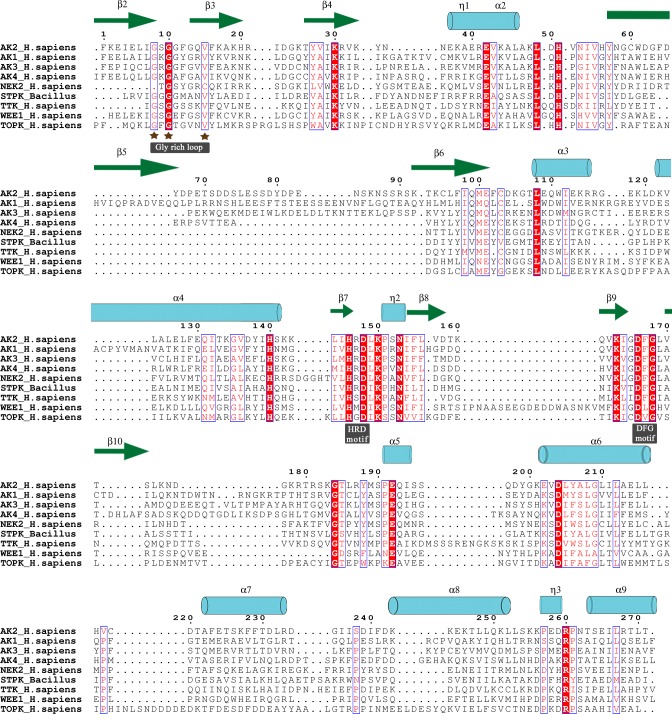
MSA of EIF2AKs. Conservation plot of EIF2AK alignment is shown in alignment plot with out-group sequences of NEK2, TTK, WEE1, TOPK and STPK. Identical residues are highlighted in red background and similar residues are colored red. PDB structure 2A1A of human EIF2AK2 kinase domain is used as structural reference. The α-helices, β-strands and 3_10_-helix are rendered with arrows, medium and small squiggles and numbered with the symbols α, β and η symbols.

Four sub-families of EIF2AK exhibit homology with overall average sequence identity of 41%. Alignment file shows conserved sequence patterns of glycine rich loop GxGxxGxV, in EIF2AK sequence family, which interacts with phosphate moiety of ATP. In outgroup sequences and prokaryotic STPK sequences motif is variant with high substituted third Gly and ultimate Val residues. Conserved Asp residue of N-terminal end HRD[LI]KPxN motif stabilizes pronated form of transferred phosphate moiety of ATP molecule. In distinct related protein sub-families like TTK, WEE1 and TOPK Arg residue is substituted with Ser, Met and Gly respectively. Similarly DFG motif interacting with Mg^2+^ cofactor is highly conserved in serine threonine kinases but is highly substituted in WEE1 and TOPK. An average sequence identity of 68%, 56%, 70% and 48% is found in EIF2AK subgroups, which was calculated using ALISTAT server[54]. Alignment of subfamily sequences shows a similar conservation of above mentioned sites, as they are primarily engaged in phosphorylation of eIF2α. Conservation of residues in alignment is plotted with reference sequences using ESPript 3.0 server[55] (Fig 3).

### Gene structure and order analysis

**Fig 4:**
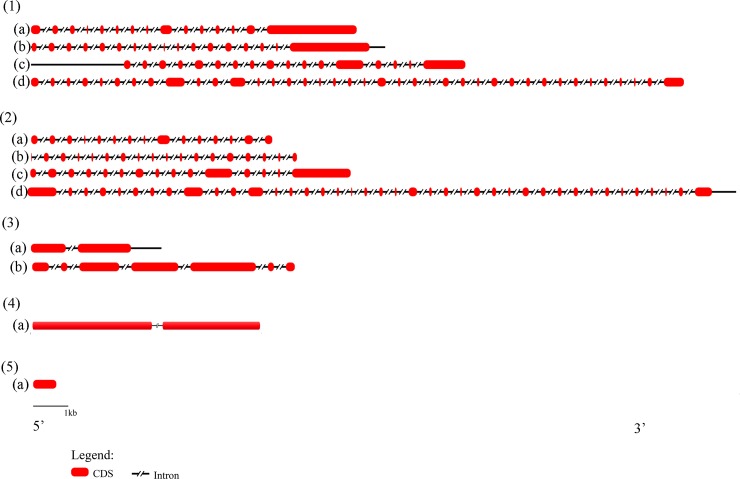
Gene structure of EIF2AKs. Figure showing number of introns and exons of EIF2AK genes. (1)(a) to (1)(d) show intron exon map of EIF2AK1, EIF2AK2, EIF2AK3 and EIF2AK4 of *Homo sapiens* with exon numbers 15, 17, 17, 39. Similarly (2)(a) to (2)(d) show intron exon map of *Xenopus tropicalis’* EIF2AK1, EIF2AK2, EIF2AK3 and EIF2AK4 with 15, 18, 15 and 40 exons. 3 (a), (3)(b) show intron exon maps of *Musca domestica*’s EIF2AK3, EIF2AK4 with a fewer exons 2 and 7. (4)(a) shows gene structure of *Saccharomyces cerevisiae* with two exons. (5)(a) shows gene structure of STPK from *Bacillus subtilis* with single exon. Legend shows the representation of CDS and intronic regions.

Orthologous genes from completely sequenced genomes share a conserved exon–intron structure through evolutionary lineage[56–58]. A comparative analysis of gene structures of four EIF2AK sub-families was adopted (Fig 4). Four members of EIF2AK family show an altered gene structure in subgroups as well as within subgroups during course of evolution. EIF2AK1 gene is encoded by 15 exons whereas EIF2AK2 gene is encoded by 17–18 exons that remain unvaried in chordate evolution. EIF2AK3 is coded by 15–22 exons, which alters, based on class of evolution. Larger number of exons of 39–40, code EIF2AK4 gene that is the largest of all EIF2AK family sequences. EIF2AK3 and EIF2AK4 of invertebrates are coded by 4 and 10 exons, which show a conservation in numbers. The few intron loss or gain events that occur in these sequences seem to be dependent on lineage-specific event. Higher conservation of exon and coding position show evolutionary conservation of EIF2AK family.

**Fig 5:**
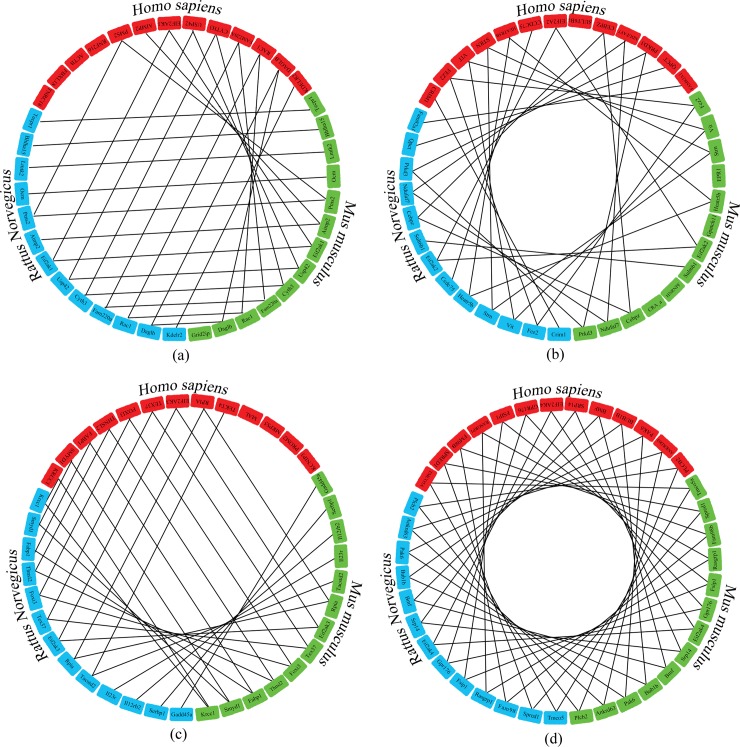
Gene order in EIF2AKs. Comparison of gene conservation surrounding the (a) EIF2AK1, (b) EIF2AK2, (c) EIF2AK3 and (d) EIF2AK4 genes. Genes from *Homo sapiens*, *Rattus norvegicus* and *Mus musculus* are indicated by red, blue and green colors respectively. Lines joining similar genes show the conservation of gene order across above species occurring close to EIF2AK genes.

Synteny is conservation of blocks of order within two sets of chromosomes that are being compared with each other. EIF2AK sequences show a conserved sequence in closely related chordates *Homo sapiens*, *Mus musculus* and *Rattus norvegicus* (Fig 5). EIF2AK1, EIF2AK2, EIF2AK3 family sequences show a conservative order of 5–6 genes showing conserved chromosomal origin across these species. EIF2AK4 sequence which shows an evolutionary lineage in lower organisms has a high conserved gene order in above mentioned species with 11 sequences conserved. Gene order in lower non-chordates is highly variable and shows a little conservation due to less number of genes they code.

### Selection pressure

**Fig 6:**
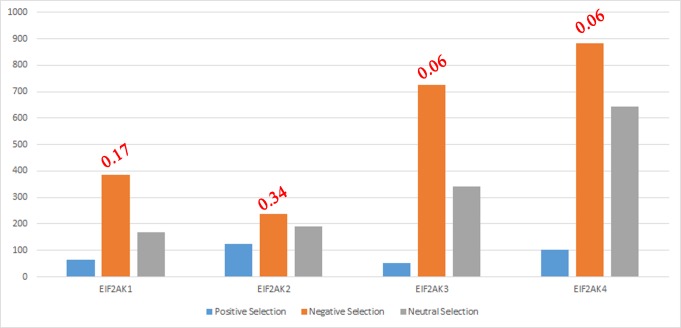
Number of sites under natural selection. Bar chart showing the number of sites under positive selection, negative selection and neutral selection. ω value of each sub-family is shown at the top of bar.

Selection pressure acting on four EIF2AK sub-families is estimated to determine type of natural selection acting on these genes (Fig 6). EIF2AK genes that show an increased number of sites are subjected to negative selection, which aids in the removal of deleterious alleles. EIF2AK1, EIF2AK3 and EIF2AK4 genes show an increase in number of sites under an increased negative selection. EIF2AK2 gene not only shows a fall in numbers of the sites under negative selection but also an increase in number of sites under positive selection. Due to rapid increase in positive selection site numbers of EIF2AK2, gene shows an increased ω value of 0.35 compared to other EIF2AK’s ω value ranging from 0.06–0.17. These studies show that EIF2AK2 genes involved in host-pathogen interactions are subjected to an intense positive selection. Leigh Van Valen’s Red Queen hypothesis explains the evolutionary dynamics of host–pathogen interactions[59]. It hypothesizes the arms race of coevolution between two intensely competing species to gain advantage over their rival, leading to evolutionary stasis in the long term, whereby the two species continue to coexist. EIF2AK2 is an example of host gene that is under higher positive selection to deny access to the pathogen, which constantly devises novel strategies for evading respective host’s defensive systems[60].

### Functional divergence of KD

Amino acid residues are said to be functionally important if it they are evolutionarily conserved. Putative changes in evolutionary conservation of amino acids at a particular residue may indicate its involvement in functional divergence. KD of four EIF2AKs have same functional role of protein synthesis inhibition, through eIF2α phosphorylation. However, KD of these proteins also has altered functions of phosphorylation of varied substrates like p53/TP53, PPP2R5A, DHX9, ILF3, IRS1 and have specific evolved inhibitors like TAT, K3L etc. Type I functional divergence refers to evolutionary process resulting in site-specific rate shifts after gene duplication, which alters highly conserved amino acid in one duplicate gene to a highly variable in other one. Type II functional divergence results in a site-specific property shift. Two evolutionally conserved orthologous genes homologous residues are subjected to a radical shift of amino acid property e.g., positively vs. negatively charged have occurred between two duplicate genes; otherwise, they are both evolutionarily conserved within each of orthologous genes. The coefficient of functional divergence between duplicate genes is defined as probability of being linked to functional divergence, with a higher value indicating a high level of divergence. Furthermore, a site-specific profile based on empirical posterior analysis can be used to predict residues that are liable to be responsible for functional divergence between two clusters.

S2 Table contains information of type I and type II sites, with posterior probability cutoff determined by a steep fall in the values (S1 Fig), along with residues from pairs of protein. In present study, a set of 72 EIF2AK sequences consisting of four sub-families along with closely related WEE1 and distinctly related STPK’s of bacterial origin is used to estimate coefficient of type I and type II functional divergence among pairs of these families and sub-families. Estimates of coefficient of divergence (θ) signify occurrence of both type I and type II divergence in KD of four EIF2AK proteins along with WEE1 and STPK. θ_I_ values for proteins range from 0.167 to 0.894 showing a little occurrence of type I functional divergence. Forty putative type I divergence sites are predominantly lying on C lobe of KD, are identified with posterior probability above the cutoff. Protein sequences show less incidence of type II divergence with a putative number of seventeen sites in MSA falling under this category. A few sites merely occur in N lobe and are widespread in C lobe. θ_II_ values for EIF2AK proteins range from 0.011 to 0.755 with a higher standard error values to diversified amino acids. Majority of these sites lie in the organized structural regions of KD whose substations impart an altered function.

Family pairs show a higher occurrence of type I divergence sites than type II divergence sites between pairs of sub-families, showing high substitution rates with a conservation of aa features (S3 Table). This is evident by a higher level of purifying selection acting on these sites. A few sites type I and type II divergence are observed in EIF2AKs, showing a conservation of KD. EIF2AKs show an increased occurrence of type I divergence with closely related WEE1 sequences than STPK of earlier origin, which predicts earlier diversification of EIF2AKs in lower organisms. Inter family comparison of EIF2AKs along with WEE1 and STPK shows fewer occurrences of type II divergent sites, which show evolutionary conservation of KD. Unique type I divergent values of EIF2AK4 sequences in comparison with other EIF2AKs illustrate its primitive origin in lower multicellular forms and its close conservation with STPK manifest origin of EIF2AKs from bacterial serine threonine kinases.

Altered interactions of proteins can be analyzed by functional divergence studies on binding site residues. Substrate protein eIF2α[61] and inhibitor protein K3L[62] binding sites are analyzed for significant functional divergence (Table 1). In regards to eIF2α; EIF2AKs do not show a significant functional divergence sites whereas non-interacting proteins like WEE1 and STPK show significant number of sites with type II divergence. Nonspecific inhibitor protein K3L has sites with significant functional divergence across EIF2AK family as well as WEE1 and STPK families. Common binding site 491 shows a significant number of type II divergence unlike other binding sites, which is asserted by selection pressure of both eIF2α and K3L proteins. Thus, binding sites of EIF2AK are under a constant selection pressure, which substitute functional site residues with drastic chemical variation of type II divergence altering interaction mechanisms to increase binding abilities.

**Table 1:** Functional divergence of binding sites. Type I and Type II divergence values for substrate eIF2α and inhibitor K3L binding sites on PKR. The significant values of divergent sites are highlighted in bold.

Residues	Alignment position	EIF2AK1		EIF2AK3		EIF2AK4		WEE1		STPK	
		θ_I_	θ_II_	θ_I_	θ_II_	θ_I_	θ_II_	θ_I_	θ_II_	θ_I_	θ_II_
**eIF2α binding site**											
**451**	375	0.116	0.000	0.079	0.000	0.106	0.000	0.795	**5.913**	0.474	0.000
**452**	376	0.280	0.166	0.082	0.144	0.108	0.576	0.532	**0.884**	0.357	**1.370**
**453**	377	0.497	0.501	0.383	0.157	0.276	0.632	0.438	**0.856**	0.217	**0.849**
**484**	420	0.638	0.473	0.183	0.597	0.210	0.318	0.483	0.216	0.658	0.470
**490**	426	0.116	0.000	0.079	0.000	0.143	0.000	**0.971**	0.334	0.477	**1.602**
**491**	427	0.378	**1.149**	0.361	**2.218**	0.169	**0.933**	0.724	**1.186**	0.289	0.634
**K3L binding site**											
**405**	268	0.116	0.000	0.617	1.032	0.141	0.000	0.459	0.000	0.499	0.734
**455**	379	0.169	**2.290**	0.079	0.000	0.566	0.617	0.795	**1.565**	0.598	0.000
**473**	408	0.679	0.426	0.125	0.287	0.202	**1.673**	0.677	0.000	0.234	**1.555**
**480**	415	0.116	0.000	0.079	0.000	0.105	0.000	0.459	0.000	0.138	0.000
**491**	427	0.378	**1.149**	0.361	**2.218**	0.169	**0.933**	0.724	**1.186**	0.289	0.634
**504**	451	0.187	0.271	0.346	0.000	0.532	0.000	0.536	0.000	0.316	0.000

## Conclusion

A large number of putative EIF2AK sequences were identified and classified in lower non-chordates, which show primitive origin of these genes. Comparative analysis at amino acid levels and gene structures supported orthologous origin of KD from bacterial PknB and it might have gained diversified signaling domains through events like chromosomal crossover. Phylogenetic studies show a separation of EIF2AK families into distinct clades and subclades, which show orthologous origin of these genes. All four genes are subject to a purifying selection, but EIF2AK2 shows an increased ω value due to its viral interactions. Functional divergence shows fewer sites with type I divergence and type II divergence sites with a conservation in KD structural features. Cluster specific analysis tells variation of functional divergence sites of closely related EIF2AKs with distinctly related families of WEE1 and STPK. Altered interactions of KD with substrates like eIF2α and K3L, which activates novel signaling cascade reactions, can be accountable to functional divergence, which imparts diversity and specificity in recognizing interacting proteins to KD. Site directed mutagenesis studies of proposed divergence sites would aid in understanding anonymous functions of KDs.

## Supporting information

S1 Table EIF2AK sequences identified. List of EIF2AK sequences identified by homology based searching. The representative sequence names used in analysis, source organism, phyla and NCBI Gi number of sequence are shown in table. The 852 sequences retrieved were tabulated with above columns.
(XLS)

S2 Table Divergence of EIF2AK’s. The residues posterior probability of the type I and type II divergence with the values greater 0.95 is shown in the table (a) and table (b) respectively. Residual position of the sites in alignment and in human EIF2AK sequences along with altered residues is also shown.
(XLS)

S3 Table Type I and type II divergence between pairs of sequences. The θ value indicates extent of divergence and SE indicates standard error. Number of sites across KD are respectively indicated.
(XLS)

S1 Fig Graphs showing cutoff values of (a) Type I and (b) Type II divergence. The cutoff is indicated by a line.
(XLS)
